# A randomized, crossover study of the acute effects of acarbose and gastric distension, alone and combined, on postprandial blood pressure in healthy older adults

**DOI:** 10.1186/s12877-019-1251-7

**Published:** 2019-08-30

**Authors:** Hung Pham, Laurence Trahair, Liza Phillips, Christopher Rayner, Michael Horowitz, Karen Jones

**Affiliations:** 1 NHMRC Centre of Research Excellence in Translating Nutritional Science to Good Health, Adelaide Medical School, The University of Adelaide, Level 5 Adelaide Health and Medical Sciences Building, Cnr North Tce and George St, Adelaide, SA 5005 Australia; 2 Endocrine and Metabolic Unit, Royal Adelaide Hospital, Adelaide, Australia; 3 Gastroenterology and Hepatology Unit, Royal Adelaide Hospital, Adelaide, Australia

**Keywords:** Postprandial hypotension, Acarbose, Gastric distension

## Abstract

**Background:**

Postprandial hypotension (PPH) occurs frequently in the elderly and patients with type 2 diabetes, and lacks a satisfactory treatment. Gastric distension and the α-glucosidase inhibitor, acarbose, may attenuate the postprandial fall in blood pressure (BP) by complementary mechanisms. We aimed to determine whether gastric distension and acarbose have additive effects to attenuate the fall in BP induced by oral sucrose.

**Methods:**

Ten healthy older adults (74.0 ± 1.4 yr) had measurements of BP and superior mesenteric artery (SMA) blood flow for 120 min after receiving either (i) the ‘study drink’ of 100 g sucrose in 300 mL of water (control treatment), (ii) a 300 mL water ‘preload’ 15 min before the ‘study drink’ (distension treatment), (iii) 100 mg acarbose dissolved in the ‘study drink’ (acarbose treatment) or (iv) a 300 ml water ‘preload’ 15 min before 100 mg acarbose dissolved in the ‘study drink’ (acarbose and distension treatment).

**Results:**

The area under the curve (AUC)_0–120min_ for mean arterial pressure (MAP) was greater (*P* = 0.005) and the maximum fall in MAP was less (*P* = 0.006) during treatments with acarbose. Gastric distension did not affect the MAP-AUC_0–120min_ response to acarbose (*P* = 0.44) and there was no effect of gastric distension alone (*P* = 0.68). Both acarbose treatments attenuated the rise in SMA blood flow (*P* = 0.003), whereas gastric distension had no effect.

**Conclusions:**

In healthy older adults, acarbose (100 mg), but not gastric distension, attenuates the fall in BP and rise in SMA blood flow after oral sucrose. The observations support the use of acarbose, but not gastric distension, to attenuate a postprandial fall in BP.

**Trial registration:**

The study was retrospectively registered at (ACTRN12618000152224) on February 02nd 2018.

## Background

Postprandial hypotension (PPH), usually defined as a fall in systolic blood pressure (BP) of ≥20 mmHg, within 2 h of a meal [1, 2], occurs frequently, e.g. in 24–38% of ‘healthy’ older adults [3] and 25–40% of patients with type 2 diabetes (T2DM) [2, 4], and is associated with major adverse sequelae, including syncope and falls [1, 2], as well as increased mortality [3]. Current management is suboptimal [4].

The pathophysiology of PPH is heterogeneous, which may account for the lack of effective management. Multiple factors appear to be involved, including autonomic dysfunction, the release of gastrointestinal hormones, meal composition, gastric distension, and small intestinal nutrient delivery. After a meal, there is substantial splanchnic blood pooling [2], and in healthy young individuals, baroreflex mechanisms protect against a post-meal decrease in BP through compensatory increases in heart rate (HR), stroke volume and cardiac output [5]. In ‘healthy’ older adults and in patients with PPH, these compensatory responses are inadequate to maintain BP [2]. We have shown in healthy older adults and people with T2DM that the magnitude of the postprandial fall in BP is greater when the rate of gastric emptying [6], or direct small intestinal nutrient delivery [7, 8], is relatively more rapid. For example, when glucose is infused into the duodenum at rates spanning the normal physiological range, a 3 kcal/min load induces a much greater decrease in BP and rises in HR and superior mesenteric artery (SMA) blood flow than 1 kcal/min [8]. Moreover, PPH is associated with more rapid gastric emptying [9]. In contrast to the effect of small intestinal nutrient delivery, gastric distension appears to be protective in PPH. Drinking water with a meal has been reported to attenuate the postprandial fall in BP in healthy older adults [10, 11], as well as in patients with autonomic failure [10–12], probably primarily by inducing gastric distension. For example, Gentilcore et at (2008) reported that gastric distension with water at a volume as low as 300 mL diminished the fall in BP in response to intraduodenal glucose in the healthy elderly [13]. The pressor response to water may also be greater in patients with autonomic failure [11].

The alpha-glucosidase inhibitor, acarbose, is used widely in the management of T2DM. By delaying intestinal disaccharide absorption, it reduces postprandial glycemia [14]. Acarbose also stimulates the release of the incretin hormone, glucagon-like peptide-1 (GLP-1) [15], and slows gastric emptying [16], which may contribute to glucose lowering in T2DM. We reported previously that acarbose (100 mg) attenuated the fall in systolic BP induced by oral sucrose in healthy older adults, an effect associated temporally with slowing of gastric emptying and stimulation of GLP-1 [17]. However, when administered intraduodenally i.e. bypassing any effect of slowing gastric emptying, acarbose also attenuated the fall in BP induced by sucrose [18], which may reflect an effect of acarbose to reduce splanchnic blood flow [18]. Subsequent studies support the efficacy of acarbose in the management of PPH to attenuate [19, 20], but not abolish, the fall in BP, although these have, for the main part, employed small cohorts and associated with substantial methodological limitations. Somewhat surprisingly, only one study has hitherto evaluated the effect of acarbose (50 mg) on the splanchnic blood flow response to a meal [21], which appears central to the hypotensive response. Furthermore, this study was in a heterogeneous group of individuals with PPH and was neither randomised nor blinded in design, compromising meaningful interpretation [21].

While it is apparent that gastric distension (induced by water drinking) and acarbose theoretically have complementary effects to attenuate the postprandial fall in BP, this has hitherto not been evaluated, which is surprising given the simplicity and safety of both approaches. The aim of this study was to determine the acute effects of water drinking and acarbose, alone and combined, on the BP and SMA blood flow responses to oral sucrose. To establish ‘proof-of-principle’, we studied a cohort of healthy older adults, rather than patients with PPH.

## Methods

### Subjects

Ten healthy older adults living in the community (2 male, and 8 female, mean age 74.0 ± 1.4 years, BMI 26.2 ± 1.1 kg/m^2^) were recruited by advertisements placed around the hospital, in newspapers and via social media, and from a database of subjects who had participated in research studies and consented to be contacted. All healthy subjects were non-smokers and none had a history of gastrointestinal disease or surgery, diabetes, significant respiratory, renal or cardiac disease, chronic alcohol abuse, epilepsy, or was taking medication known to influence BP or gastrointestinal function. The study was conducted in accordance with the Declaration of Helsinki following the provision of written informed consent by each of the participants. The study was registered at http://www.ANZCTR.org.au (ACTRN12618000152224) and approved by the Human Research Ethics Committee of the Royal Adelaide Hospital. The study adheres to CONSORT guidelines.

### Protocol

**Fig. 1:**
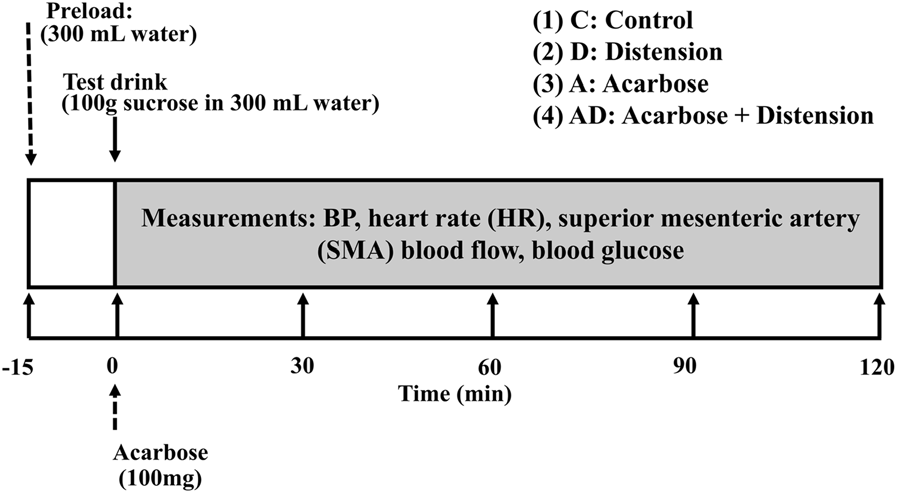
Schema of the study protocol

Each subject was studied on 4 occasions, separated by at least 1 week, in a randomized, crossover design. On each study day, the subject attended the University of Adelaide, Discipline of Medicine at the Royal Adelaide Hospital at ∼ 09.00 h after an overnight fast (14 h for solids; 12 h for liquids) [22]. Subjects were seated in a chair, an intravenous cannula was inserted into an antecubital vein for blood sampling and an automated BP cuff placed around the opposite arm. Following a period of ‘rest’ of 15–30 min to allow baseline BP to stabilise [7], subjects were given, in random order, either the (i) control treatment (C): a test drink comprising 100 g sucrose dissolved in 300 mL of water (~ 407 kcal), (ii) distension treatment (D): a ‘preload’ of 300 mL water 15 min before the ingestion of the test drink, (iii) acarbose treatment (A): 100 mg acarbose (Glucobay™, Bayer Schering Pharma AG, Berlin, Germany) dissolved in the test drink or (iv) combined treatment (AD): the water preload was given 15 min before the ingestion of acarbose which was dissolved in the test drink. Both the ‘preload’ and test drink were consumed within 2 min with time zero (t = 0 min) defined as the end of test drink ingestion (Fig. 1).

Measurements of BP, HR and SMA blood flow were performed at regular intervals until t = 120 min, as the greatest fall in postprandial BP is known to occur within that time [3]. At the end of each study day, each subject was provided with lunch and a final BP measurement was taken prior to them leaving the laboratory.

### Measurements

#### Blood pressure and heart rate

Systolic and diastolic BP (SBP and DBP) and HR were measured with an automated oscillometric BP monitor (DINAMAP ProCare 100, GE Medical Systems, Milwaukee, WI, USA*)* at 3-min intervals prior to ingesting the test drink, and, subsequently, every 3 min between t = 0–120 min [6, 8, 13, 17, 23–29]. An average of BP and HR measurements obtained at t = − 24, − 21, − 18 min prior to the ingestion of the preload (t = − 15 min), or, on the study day without a preload, at t = − 9, − 6, − 3 min prior to ingestion of the test drink, were calculated to represent baseline BP and HR. The mean arterial pressure (MAP) was calculated using the formula MAP = DBP+[(SBP-DBP)/3]. PPH was defined as a fall in systolic BP of at least 20 mmHg that was sustained for 30 min or more [6].

#### Superior mesenteric artery blood flow

SMA blood flow (mL/min) was measured prior to the ingestion of the preload (t = − 17 min) and/or test drink (t = − 2 min), and then every 15 min between t = 0–120 min using a Logiq e™ ultrasound system (GE Healthcare Technologies, Sydney, NSW, Australia) incorporating a 3.5C broad spectrum 2.5–4 MHz convex linear array transducer [30].

#### Blood glucose concentrations

Venous blood samples (~ 15 mL) were drawn immediately before ingestion of the preload (t = − 17 min) and /or test drink (t = − 2 min) and then at t = 30, 60, 90 and 120 min. Blood glucose concentrations were determined during the study using a portable glucometer (Medisense Companion 2 Meter, Medisense Inc., Waltham, MA, USA) [31].

#### Autonomic nerve function

Autonomic nerve function was assessed on one of the 4 days using standardized cardiovascular reflex tests with an appropriately sized BP cuff in a temperature-controlled, quiet, clinical study room (ANX-3.0 autonomic monitoring system (ANSAR Medical Technologies, Inc., Philadelphia, PA)) [32–34]. Testing was performed in the fasting state, preceded by 10–15 min of rest in the recumbent position. Parasympathetic function was evaluated by: i) the variation (R-R interval) of the HR during deep breathing whereby 6 respiratory cycles were performed and E/I ratio calculated from the peak expiratory and peak inspiratory heart beat intervals and ii) the response to standing (30:15 ratio), with the relevant ECG parameters captured and analyzed electronically [32]. Sympathetic function was assessed by the fall in systolic BP in response to standing whereby baseline BP was taken following 10–15 min in a semi-recumbent position immediately and 3 min after standing. An abnormal BP response to standing was defined as a decrease in BP of 20/10 mmHg or more upon standing over resting. Each of the test results was scored according to predefined criteria that were adjusted for age where 0 was considered normal, 1 as borderline, and 2 as abnormal providing a total maximum score of 6 [34]. A score of at least 3 was considered to suggest autonomic dysfunction.

### Statistical analysis

The maximum fall in MAP and rise in HR were defined as the greatest change from baseline in each subject at any given time point for each treatment. Areas under the curve (AUCs) were calculated using the trapezoidal rule from t = 0–120 min for MAP and HR, and from t = − 2–120 min for SMA blood flow and blood glucose. Repeated measures two-factor ANOVA was used to evaluate the effects of acarbose, gastric distension and their interaction for the AUCs for MAP, HR, SMA blood flow and blood glucose. All analyses were performed using SPSS version 23 (SPSS, Chicago, IL, USA). MAP and HR are presented as change from baseline values and SMA blood flow and blood glucose concentrations as absolute values. Data are presented as mean values ± SEM, unless stated otherwise. A *P* value < 0.05 was considered significant in all analyses.

## Results

The studies were reasonably well tolerated. Flatulence and/or diarrhea were reported by 3 of the 10 subjects on acarbose days; in each case the onset of symptoms was about 3–4 h after ingestion. Three subjects fainted during the study (2 subjects on the distension day and 1 on the acarbose day between t = 60–75 min), which, in each case, resolved promptly after lying supine for 15–30 min. In 1 of the 3 subjects, fainting was concordant with PPH on the distension day. No subject had autonomic neuropathy (mean score: 1.4 ± 0.2). In 1 subject, the ultrasound images of SMA blood flow were suboptimal on all study days due to the presence of bowel gas, precluding their use. In another subject, insertion of the intravenous cannula for blood sampling was unsuccessful on all 4 study days due to poor venous access. Accordingly, BP and HR data are available in 10, while SMA blood flow and blood glucose data are available in 9, subjects.

### Blood pressure and heart rate

There were no differences in baseline BP or HR among the 4 treatments (control vs. distension vs. acarbose vs. combined treatment, respectively): SBP (133 ± 3.7 mmHg vs. 128 ± 2.8 mmHg vs. 125 ± 3.6 mmHg vs. 129 ± 3.7 mmHg; *P* = 0.19); DBP (72 ± 2.6 mmHg vs. 73 ± 2.8 mmHg vs. 70 ± 1.9 mmHg vs. 73 ± 1.7 mmHg; *P* = 0.23); MAP (92 ± 2.2 mmHg vs. 91 ± 2.4 mmHg vs. 88 ± 2.1 mmHg vs. 92 ± 1.9 mmHg; *P* = 0.18) and HR (67 ± 2.1 beats/min vs. 68 ± 2.5 beats/min vs. 66 ± 2.8 beats/min vs. 67 ± 2.6 beats/min; *P* = 0.76). One subject on the control day and another on the distension day had PPH. No subject experienced PPH on either of the acarbose study days.

#### Mean arterial pressure

**Fig. 2:**
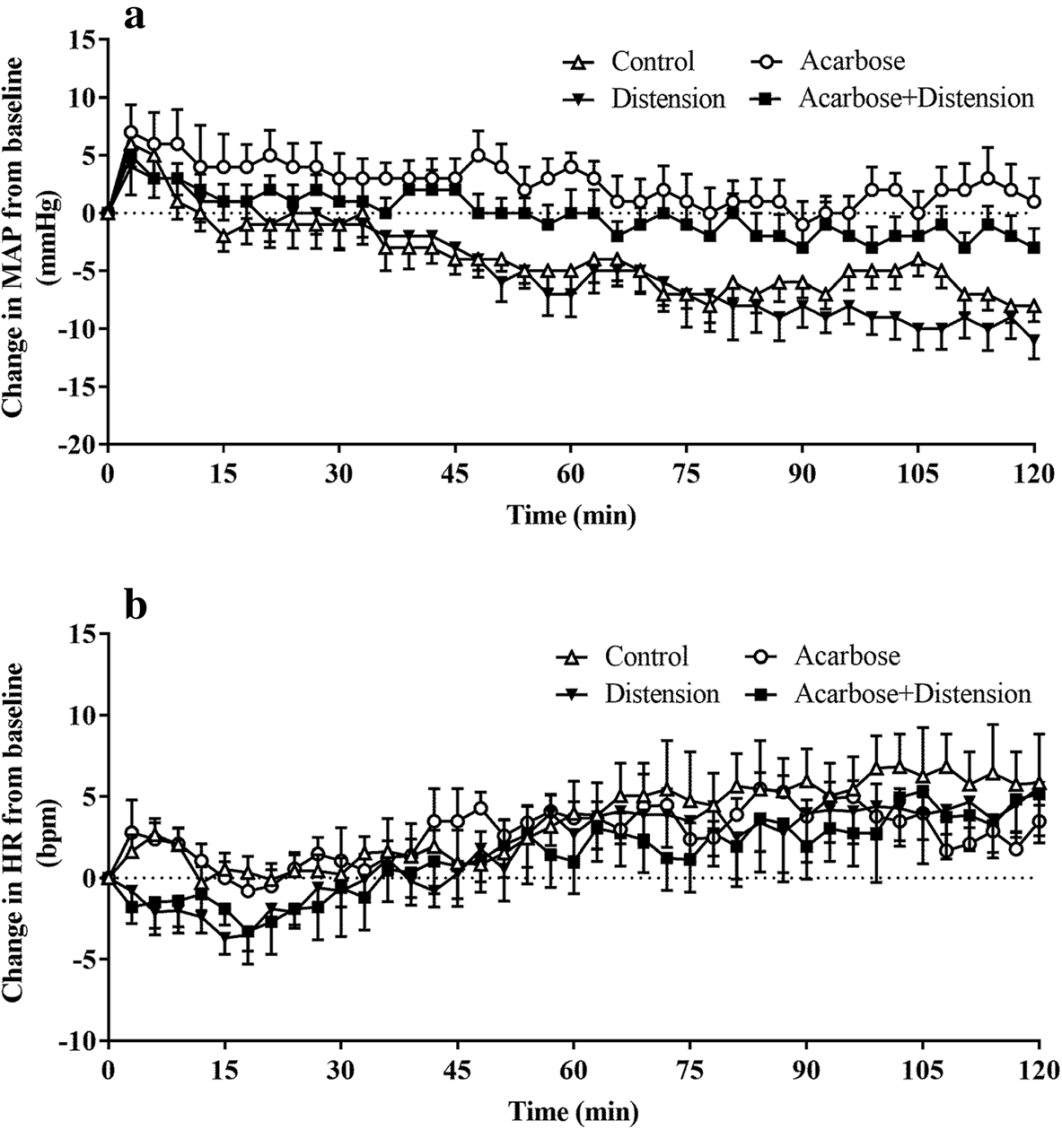
Changes in mean arterial pressure (MAP) (**a**), and heart rate (HR) (**b**) from baseline in response to control (C), distension (D), acarbose (A) and combined (AD) treatments. Data are mean values ± SEM (*n* = 10). The AUC_0–120_ for MAP was greater (A and AD compared to C; *P* = 0.005) with both acarbose treatments with no difference between C and D. The AUC_0–120_ for HR was less (A and AD compared to C; *P* = 0.04) with both acarbose treatments with no difference between C and D

Between t = 0–120 min, there was a fall in MAP during control and distension treatments (*P* < 0.001 for both), but no overall change during either acarbose (*P* = 0.44), albeit a non-significant trend for combined (*P* = 0.06), treatments. There was a treatment effect for acarbose, so that the AUC_0–120min_ for MAP was greater during treatments with acarbose (A: 10,625 ± 237 mmHg.min and AD: 10,721 ± 232 mmHg.min; *P* = 0.005) compared to control (C: 10,366 ± 281 mmHg.min), but not for distension (D: 10,106 ± 252 mmHg.min; *P* = 0.68). There was also no interactive effect between acarbose and distension on the AUC_0–120min_ for MAP (*P* = 0.44) (Fig. 2a).

The maximum fall in MAP from baseline was less during treatments with acarbose (*P* = 0.006) (acarbose: − 6.9 ± 1.8 mmHg and combined treatment: − 8.2 ± 1.5 mmHg) compared with control (− 12.2 ± 1.4 mmHg). There was no effect of gastric distension alone (− 15.3 ± 1.8 mmHg, *P* = 0.21) and no difference between the acarbose treatments with or without gastric distension (*P* = 0.58).

#### Heart rate

Between t *=* 0–120 min, there was a rise in HR among 4 treatments (*P* < 0.005 for all) (Fig. 2b). There was a treatment effect for acarbose, so that the AUC_0–120min_ for HR was lower during treatments with acarbose (A: 8057 ± 356 bpm.min and AD: 7985 ± 315 bpm.min; *P* = 0.04) compared to control (C: 8252 ± 357 bpm.min), but not for distension (D: 8165 ± 312 bpm.min; *P* = 0.55). There was also no interaction between acarbose and distension on the AUC_0–120min_ for HR (*P* = 0.92) (Fig. 2b).

There was no treatment effect for the maximum rise in HR from baseline among the 4 treatments, so that there was no significant difference in the maximum rise in HR during acarbose (10.4 ± 1.4 bpm, *P* = 0.93), distension (10.5 ± 1.9 bpm, *P* = 1.0) and combined treatments (10.6 ± 2.4 bpm, *P* = 0.88), compared to control (10.7 ± 2.5 bpm).

### Superior mesenteric artery blood flow

**Fig. 3:**
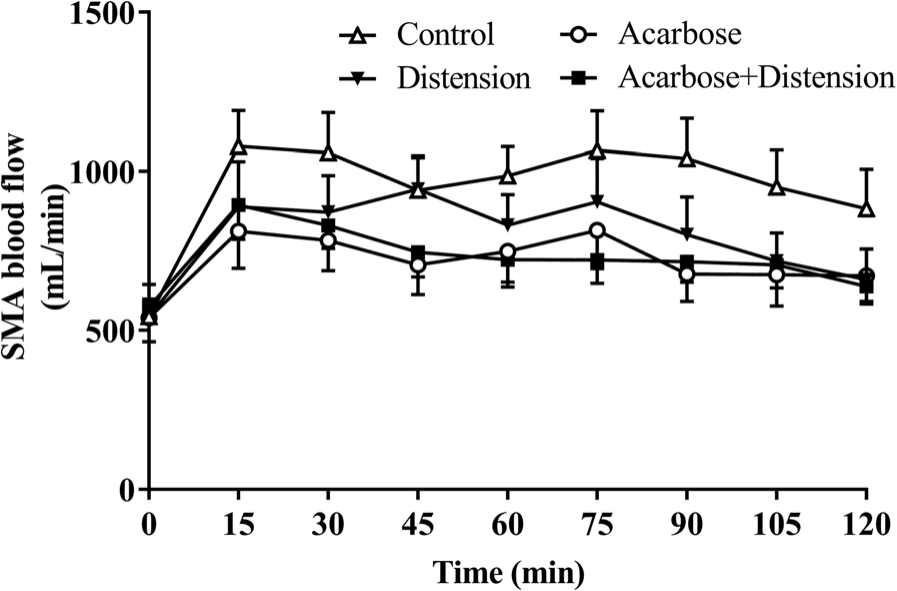
Superior mesenteric artery blood flow in response to control (C), distension (D), acarbose (A) and combined (AD) treatments. Data are mean values ± SEM (*n* = 9). The AUC_0–120_ for SMA flow was less (A and AD compared to C; *P* = 0.003) with both acarbose treatments with no difference between C and D

There was no difference (*P* = 0.83) in baseline (t = − 2 min) SMA blood flow among the 4 treatments (control vs. distension vs. acarbose vs. combined treatment: 543 ± 55 mL/min vs. 583 ± 91 mL/min vs. 538 ± 75 mL/min vs. 579 ± 50 mL/min, respectively) (Fig. 3).

Between t = − 2–120 min, there was a rise in SMA blood flow with the control (*P* < 0.001) and distension (*P* = 0.02), and a trend for an increase after acarbose alone (*P* = 0.08) and combined (*P* = 0.07), treatments (Fig. 3). There was a treatment effect for the AUC_0–120min_ of SMA blood flow for acarbose, so that SMA blood flow was less during acarbose treatments, with or without distension (A: 86,689 ± 10,725 mL/min.min and AD: 87,720 ± 6750 mL/min.min; *P* = 0.003), compared with control (C: 111,738 ± 12,631 mL/min.min). There was no difference between distension (D: 95,846 ± 11,418 mL/min.min) and control (*P* = 0.41), and no additive effect between acarbose and distension in the combined treatment (*P* = 0.15) (Fig. 3).

The maximum rise in SMA blood flow during acarbose treatments (acarbose: 963 ± 123 mL/min and combined treatment: 983 ± 102 mL/min) was less (*P* = 0.03), compared to control (1173 ± 111 mL/min). There was no difference between distension (1073 ± 140 mL/min) (*P* = 0.67) and control and no interaction between acarbose and distension (*P* = 0.42) in the maximum rise in SMA blood flow (Fig. 3).

### Blood glucose

**Fig. 4:**
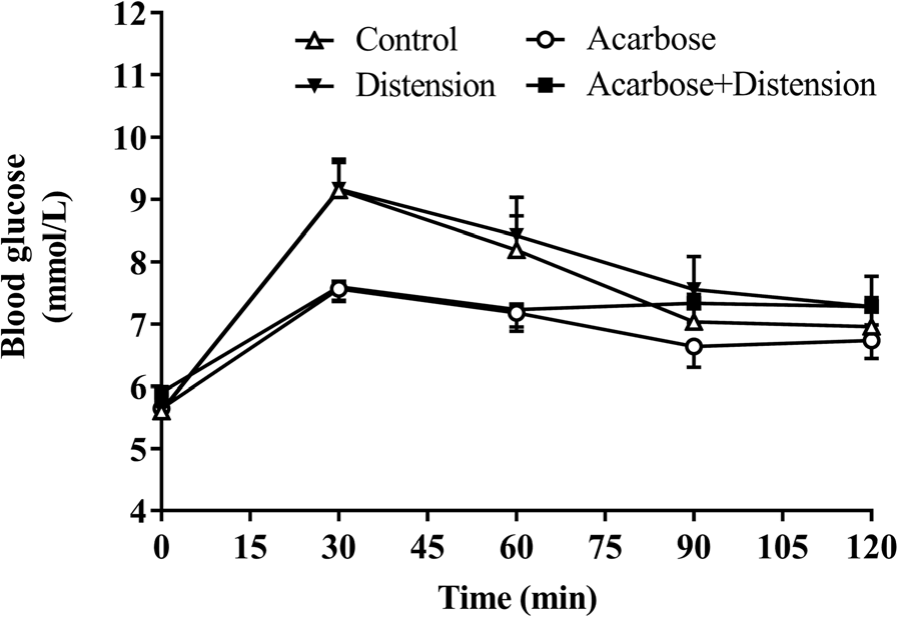
Blood glucose concentrations in response to control (C), distension (D), acarbose (A) and combined (AD) treatments. Data are mean values ± SEM (*n* = 9). The AUC_0–120_ for blood glucose was reduced (A and AD compared to C; *P* = 0.03) by both acarbose treatments with no difference between C and D

There was no difference (*P* = 0.56) in baseline (t = − 2 min) blood glucose among the 4 treatments (control vs. distension vs. acarbose vs. combined treatment: 5.6 ± 0.16 mmol/L vs. 5.6 ± 0.14 mmol/L vs. 5.6 ± 0.10 mmol/L vs. 5.7 ± 0.20 mmol/L, respectively) (Fig. 4).

Between t = − 2–120 min, there was a rise in blood glucose with all treatments (*P* < 0.001 for all) (Fig. 4). There was a treatment effect for the AUC_0–120min_ for blood glucose for acarbose, so that blood glucose was less during acarbose treatments with and without distension (A: 827 ± 28.9 mmol/L.min and AD: 863 ± 30.3 mmol/L.min; *P* = 0.03), compared with control (C: 919 ± 41.9 mmol/L.min). There was no difference between distension (D: 948 ± 53.9 mmol/L.min) and control (*P* = 0.12), nor any additive effect between acarbose and distension when combined (*P* = 0.92) (Fig. 4).

## Discussion

Our study evaluated the acute effects of acarbose and water drinking, alone and combined, on the BP and SMA blood flow responses to oral sucrose in healthy older adults. We demonstrated that in this group, ingestion of sucrose induced a substantial increase in SMA blood flow and fall in BP, as predicted [17, 18]. These changes were essentially abolished by acute administration of acarbose in a dose of 100 mg; which was well tolerated. In contrast, water drinking alone (300 mL, 15 min before the sucrose load) to induce gastric distension had no significant effect on either BP or splanchnic blood flow and did not modify the response to acarbose.

This is the first study to evaluate the interaction between acarbose and gastric distension on ‘postprandial’ BP based on their potential for synergetic, or additive, effects. The observed responses to acarbose are consistent with previous reports, including our own [17, 18, 21, 35], and support the concept that acarbose will be useful in the management of PPH. We have further demonstrated that acarbose causes a profound attenuation of the rise in SMA blood flow induced by oral sucrose [18, 21] which is likely to be central to its anti-hypotensive effect. Such an effect was suggested in a previous study that was methodologically flawed [21]. The reduction in the splanchnic blood flow response to oral sucrose induced by acarbose is likely to reflect mechanisms unrelated to slowing of gastric emptying per se [17], given that the increase in SMA blood flow induced by intraduodenal administration of sucrose in healthy older adults is also markedly attenuated by acarbose [18]. The stimulation of GLP-1 as a result of the presence of nutrient in the more distal intestine may be relevant. In particular, we have demonstrated in healthy older adults that intravenous GLP-1 reduces the SMA blood flow response to intraduodenal glucose [36] and that the ‘short-acting’ GLP-1 agonist, lixisenatide, prevents the fall in systolic BP and reduces the rise in SMA flow following a 75 g oral glucose load in healthy older subjects and T2DM patients [37]. However, in relation to a role for endogenous GLP-1, it would be expected that stimulation of GLP-1 would be associated with a concurrent equivalent increase in glucagon-like peptide-2 (GLP-2) [38, 39], which would be expected to stimulate SMA blood flow [40]. The reduction in plasma glucose induced by acarbose is predictably associated with a reduction in plasma insulin, which has vasodilatory properties [1].

We were surprised that water drinking had no significant effect on either BP or SMA blood flow. There are a number of potential explanations. We selected a relatively low volume (300 mL) drink to optimise tolerability, influenced by the outcome of our previous study demonstrating that this intragastric volume of water markedly attenuated the hypotensive response to intraduodenal glucose in healthy older adults [13]. With our study design, it would be anticipated that the majority of water would have been emptied from the stomach in the 15 min before the ingestion of the sucrose. Furthermore, the effects of gastric distension are known to be volume-dependent [41]. Hence, we cannot discount the possibility that a larger volume of water, given immediately before the sucrose may have been more effective. Moreover, the pressor effect of water drinking may also reflect changes in plasma osmolality [10, 11]. While there was a substantial fall in BP in response to sucrose, we studied healthy older adults, rather than patients with PPH, and none had evidence of autonomic neuropathy, although assessment of autonomic nerve function was indirect. The latter may also be of relevance to the negative outcome given that the pressor response to water appears to be exaggerated in patients with autonomic impairment [11]. It should also be appreciated that the number of subjects we studied was relatively small and there were non-significant trends for minor effects of water drinking soon after the sucrose drink. Accordingly, a type 2 error cannot be excluded. In addition, GLP-1 was not measured, which may have provided mechanistic insights into the role of acarbose in modulating postprandial BP. A strength of our study was the inclusion of splanchnic blood flow measurement.

## Conclusion

Previous studies have suggested that both acarbose and gastric distension may be potential approaches to reduce the postprandial fall in BP and our study aimed to evaluate the interaction between these interventions. The outcomes provide additional evidence to support the use of acarbose to attenuate the magnitude of the postprandial fall in BP, but not in combination with water drinking. The latter concept should, however, not be dismissed pending the outcome of further studies with greater subject numbers and particularly in patients with PPH and where water is consumed immediately prior to a meal.

## Data Availability

The datasets used and/or analysed during the current study are available from the corresponding author on reasonable request.

## Abbreviations

AUC: Area under the curve
BP: Blood pressure
DBP: Diastolic blood pressure
GLP-1: Glucagon-like peptide-1
GLP-2: Glucagon-like peptide-2
HR: Heart rate
MAP: Mean arterial pressure
PPH: Postprandial hypotension
SBP: Systolic blood pressure
SMA: Superior mesenteric artery
T2DM: Type 2 diabetes
